# Measuring the Mechanical Properties of Plant Cell Walls

**DOI:** 10.3390/plants4020167

**Published:** 2015-03-25

**Authors:** Hannes Vogler, Dimitrios Felekis, Bradley J. Nelson, Ueli Grossniklaus

**Affiliations:** 1 Institute of Plant Biology and Zurich-Basel Plant Science Center, University of Zurich, Zollikerstrasse 107, CH 8008 Zürich, Switzerland; E-Mail: grossnik@botinst.uzh.ch; 2 Institute of Robotics and Intelligent Systems, ETH Zurich, Tannenstrasse 3, CH 8092 Zürich, Switzerland; E-Mails: dfelekis@ethz.ch (D.F.); bnelson@ethz.ch (B.J.N.)

**Keywords:** cell wall, cytomechanics, turgor pressure, Young’s modulus, pollen tube, cellular force microscope

## Abstract

The size, shape and stability of a plant depend on the flexibility and integrity of its cell walls, which, at the same time, need to allow cell expansion for growth, while maintaining mechanical stability. Biomechanical studies largely vanished from the focus of plant science with the rapid progress of genetics and molecular biology since the mid-twentieth century. However, the development of more sensitive measurement tools renewed the interest in plant biomechanics in recent years, not only to understand the fundamental concepts of growth and morphogenesis, but also with regard to economically important areas in agriculture, forestry and the paper industry. Recent advances have clearly demonstrated that mechanical forces play a crucial role in cell and organ morphogenesis, which ultimately define plant morphology. In this article, we will briefly review the available methods to determine the mechanical properties of cell walls, such as atomic force microscopy (AFM) and microindentation assays, and discuss their advantages and disadvantages. But we will focus on a novel methodological approach, called cellular force microscopy (CFM), and its automated successor, real-time CFM (RT-CFM).

## 1. Why Should We Study Cytomechanics?

Cytomechanical studies gained increasing interest over the last years after having been neglected for a long time. Mechanical forces have been recognized to influence many biological processes in animal biology, such as the differentiation of stem cells [1,2,3,4], embryo development [5,6,7] or tumor invasion, to name just a few [8,9,10]. In plant sciences, special attention has been paid to the mechanical properties of the cell wall, which plays an important role in plant stability and the resistance against pathogens. Several comprehensive reviews describing available methods in plant mechanics were published recently [11,12,13,14,15] and will only briefly be summarized here.

In the first part of this review, we will provide a few examples of well-known agricultural and industrial problems that are related to the mechanical properties of the plant cell wall. However, the main focus will be on pollen tubes and the effect of alterations in the biochemical composition of their cell wall on its mechanical properties. In the second part, we will discuss different approaches to assess these properties, with a more extended description of the cellular force microscope (CFM), a novel and flexible system to measure a wide range of forces exerted by living cells. A short outlook for new directions and developments in cytomechanical studies will be given at the end.

Modern agriculture has to deal with diverse problems related to the composition and stability of the cell wall. Crop lodging, the permanent displacement of plant shoots, is a world-wide problem that causes severe harvest losses. For the U.K., it was reported that every 3–4 years, on average, 15%–20% of the wheat area lodges [16]. In individual fields, however, the lodged area can be much larger. Countermeasures to reduce the risk of lodging involve the production of dwarf varieties on the breeders’ side [17] and the application of plant growth regulators to shorten crops on the farmers’ side [18]. Unfortunately, these approaches lead to a yield reduction, even in years with favorable weather conditions when lodging is not a problem. Knowledge about the correlation between the biochemical composition and the mechanical stability of the cell wall could help to produce crops that reach the full yield potential, while being more resistant to lodging.

The mechanical properties of the leaf directly affect plant-herbivore interactions, resistance against pathogens and nutrient recycling via litter decomposition [19]. Plants regulate the interactions with insects by modulating their surfaces to facilitate or limit insect access [20]. Modification of leaf toughness alters palatability or digestibility and, thus, can be considered a defense strategy against herbivory [21]. Similar mechanisms restrict the access of microorganisms to plant tissue [22].

While increased leaf toughness provides protection against herbivorous pests, it poses a problem for digestion when used as animal feed. A reduction in lignin content, as it occurs in the brown midrib mutants of maize and sorghum [23,24,25], leads to better digestibility and consequently higher milk production in dairy cattle [26]. However, this comes at the cost of lower yield, mostly due to lodging. Nevertheless, the use of brown midrib maize for silage on dairy farms is economically profitable where normal weather conditions are such that lodging only poses a minor problem [26].

Many industrial processes depend on an optimal cell wall composition. Removal of lignin, for example, is a cost-intensive process in paper production, and the paper industry would benefit tremendously from reduced lignin contents in the processed wood. However, lignin is important for tree stability and cannot be reduced to arbitrary levels without knowing how to compensate for the loss of stability. Similarly, the bottleneck in cost-effective biofuel production is the efficient degradation of cell walls into fermentable sugars [27]. Therefore, knowledge about the contribution of individual compounds to the overall mechanical properties of the cell wall is crucial to optimize cell wall composition and to reduce the costs in these industries.

To understand the complex interactions between expanding cells within a tissue or an entire organism, we first need a profound understanding of the mechanical processes during growth at the single-cell level. Fundamentally, changes in the shape and size of a cell during morphogenesis result from the deformation and modification of the existing cell wall, as well as from the secretion and deposition of newly-synthesized wall materials.

## 2. The Mechanical Cell Wall Properties Are Defined by Its Composition

The mechanical properties of the plant cell wall are defined by its biochemical composition and the specific interactions between individual wall polymers. The primary cell wall is a complex compound, assembled from cellulose microfibrils, hemicelluloses, pectic polysaccharides and cell wall proteins. It is thought that individual microfibrils are linked via xyloglucan (XyG), the most abundant hemicellulose in dicotyledonous plants, to build a load-bearing network. In one of the cell wall models, XyG chains tether neighboring, well-spaced microfibrils by direct crosslinking, and the resulting cellulose/XyG network is embedded in a pectin matrix [28]. A second possibility under consideration is that the microfibril crosslinking is indirect. In this case, XyG polymers bind covalently to the pectin network, and the so-embedded XyG chains connect cellulose microfibrils that are in close contact via hydrogen bonds. The close vicinity of the fibrils renders the XyG between them inaccessible for endoglucanases [29]. If the first model were true, endoglucanase digestion of heat-inactivated cell walls should provoke cell wall extension in an extensometer assay. In cucumber cell walls, however, this effect was not found, which favors the second model [29], at least in this case.

Cellulose microfibrils act as the main load-bearing constituents of the cell wall. In a typical elongating cell, the microfibrils are arranged transversally around the cell, thereby providing reinforcement against growth in width and allowing preferential growth in length [30]. A passive reorientation from a transverse to a longitudinal pattern occurs during the elongation process [31,32]. However, in the tip-growing pollen tubes, the levels of cellulose are quite low, and despite their nearly perfectly cylindrical shape, transversally-oriented cellulose microfibrils have not been found [33,34]. This finding indicates that cellulose cannot be the only compound that counteracts the transverse stress, which is twice as high as the longitudinal stress [35].

*Arabidopsis thaliana* plants lacking xyloglucan xylosyltransferases (XXT) have a strong reduction of xyloglucans in their cell walls. However, even double mutants for *xxt1* and *xxt2* show only a weak growth phenotype, suggesting that xyloglucan cannot be the main load-bearing tether that holds the cellulose microfibrils together [29]. Interestingly, the same double mutants show a severe pollen tube growth phenotype leading to the loss of cell wall integrity and eventual bursting [36]. Overexpression of xyloglucan endotransglucosylase/hydrolases (XTHs), on the other hand, decreases the wall yield threshold. Since the amount of xyloglucans was not increased in XTH overexpressing plants, this may be an indirect effect, caused, for example, by alterations in cellulose deposition and crystallinity, due to changes in the cell wall matrix near nascent microfibrils [37].

Mutants that are deficient in linear pectic arabinans display increased stiffness and reduced strain in epidermal cells of *Arabidopsis* stems. Despite a reduction in pectic arabinan of 54% in the stem of *arabinan-deficient–1* (*arad1*) mutants, these plants show no altered growth phenotype [38]. The above examples illustrate that compensation mechanisms may suppress some effects of the changes in cell wall composition and that phenotypes become only visible in specific cell types or under unfavorable growth conditions. Moreover, such compensation mechanisms suggest the existence of a cell wall sensing system, which relays information from the cell wall to the interior of the cell, so it can react with regard to the synthesis of new cell wall components [39,40,41].

To get a clearer picture of the mechanical consequences caused by altered cell wall composition, it is important to quantify the resulting mechanical parameters.

## 3. Measuring Turgor Pressure

Plant cell growth is a fine-tuned interplay between turgor-driven cell expansion and the resistance provided by the cell wall. The importance of turgor pressure for plant growth has been known for a long time. Measuring it, however, was only possible via the plasmolytic method, where the osmolality of the external medium was increased until the protoplast started to detach from the cell wall [42,43]. It was only in 1968 when the first direct measurement method became available. The pressure probe is an oil-filled capillary that is directly inserted into the vacuole of the cell to be studied [44]. Initially, this technique was used to measure turgor in algae with very large cells [45,46], but continuous development made it possible to use the pressure probe also for small cells, such as stomatal guard cells [47,48].

The pressure probe is a versatile instrument, not only for measuring, but also for manipulating turgor pressure within a cell by injecting or removing a known volume of liquid. Determining the volumetric modulus in this way allows conclusions about the average cell stiffness [49,50,51]. Despite the versatile information it can provide, the pressure probe has some disadvantages. It is invasive and, therefore, not useful for repeated measurements on the same cell. Furthermore, its handling is rather complicated, which limits the number of measurements one can make in a day.

Ball tonometry was developed to overcome these difficulties. This method is not invasive, albeit indirect, and is based on the compression of individual plant cells with a large diameter sphere (50–500 µm) [52,53]. Due to the large diameter of the indenter, which is much bigger than the thickness of the cell wall, the elasticity of the cell wall itself can be neglected. Thus, turgor pressure is determined by the ratio between the known load and the contact area between the sphere and the cell surface. Turgor values measured with this method agree well with the direct pressure probe measurements.

## 4. Measuring Mechanical Cell Wall Properties

The plant cell wall is a remarkable structure that must, at the same time, be strong enough to withstand turgor pressure, yet flexible enough to allow controlled cell expansion. To solve this dilemma, a perfect coordination between the deformation and modification of the existing wall and the secretion and deposition of new cell wall material is required. The secret of these mechanical properties lies within the compound structure of the cell wall. Although the main components are well described [54], there is a lack of knowledge concerning their individual contributions to the mechanical properties of the cell wall. In the past, numerous methods were developed to assess cell wall stiffness [12]. Extensometer assays provided insight into the elasticity of isolated cell walls or entire tissues [55,56,57,58,59]. However, it is extremely difficult to perform experiments on individual living cells, and measuring local differences in mechanical cell wall properties is not possible using this approach.

A new, interesting approach to measure mechanical properties of living cells uses custom-made microfluidic chips, which are especially useful to study growing cells, such as pollen tubes. By modulating channel shape and diameter, as well as the stiffness of the surrounding walls, it is possible to draw conclusions about the pushing force and the elasticity of the elongating pollen tube [60,61,62]. As with extensometer assays, however, it is not possible to get information about local differences in the mechanical properties of the cell wall at high resolution. Techniques, such as optical or magnetic tweezers, provide extremely highly-resolved force and position data down to the single molecule level for animal cells [63,64,65]. However, their force range is limited to piconewtons, which is too low to assess the mechanical properties of plant cell walls.

A solution to overcome these limitations was provided by nano- and micro-indentation assays. A cell is placed on a stiff surface, *i.e.*, a glass slide, and probed with an end-effector that has a well-defined tip geometry. Cellular stiffness can be determined by monitoring the forces that are necessary to create small, local deformations of the cell wall. Several factors influence the measured apparent stiffness. Among these are cell parameters, such as cell wall elasticity, cell wall thickness, cell geometry and turgor pressure, as well as system parameters, for example indenter geometry or contact angle [66,67,68].

Atomic force microscopy (AFM) is frequently used in nanoindentation studies, allowing the combined acquisition of highly-resolved topographical and stiffness information. AFM measurements with sharp indenters and very shallow indentations allow the direct extraction of the elastic modulus of the cell wall from experimental data without the influence of turgor pressure. Using such a setup, it was shown that the outer cell wall of the *Arabidopsis* shoot apex was more elastic at the periphery than at the tip [69].

However, the forces that can be applied by an AFM are usually in the nanonewton range. Thus, they are too small to deform the walls of turgid cells enough to measure in-plane elasticity, which is influenced by cellulose microfibrils. Instead, these measurements are sensitive to the elastic modulus normal to the cell wall. This provides information about the gel matrix component of the cell wall, which contains mainly pectins and hemicelluloses [54]. Peaucelle and colleagues circumvented this problem by gluing a 5-µm glass bead to a stiff cantilever, which extended the range of the AFM to microindentation dimensions. Using this approach, the authors could show that cell softening in the incipient leaf primordium correlated with pectin methylesterase (PME) activity [70].

Using a microindentation approach combined with finite element method (FEM) modeling, Forouzesh and colleagues were not only able to characterize the elastic and viscoelastic properties of *Arabidopsis* leaf cell walls, but they could also extract turgor pressure values and get a rough estimate of the cell wall thickness [71]. Table 1 gives an overview of the advantages and disadvantages of selected methods used to measure cytomechanical properties.

**Table 1:** Advantages and disadvantages of mechanical characterization methods

	Force			Position				
Method	Resolution	Range		Resolution	Range	Specimen aspect ratio	High throughput	Non- destructive
Magnetic or								
Optical tweezers	✓	✗		✗	✗	✗	✓	✗
Extensometer	✓	✓		✗	✗	✗	✓	✗
Microfluidic chip	✓	✗		✗	✗	✗	✓	✗
AFM	✓	✗		✓	✓	✗	✗	✓
CFM	✓	✓		✓	✓	✓	✗	✓
RT-CFM	✓	✓		✓	✓	✓	✓	✓

## 5. Cellular Force Microscopy

**Figure 1:**
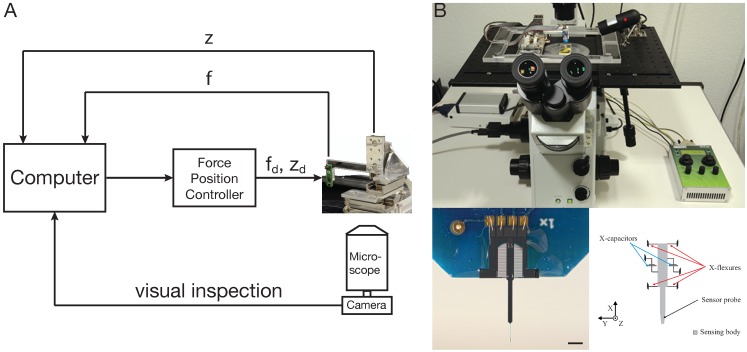
The cellular force microscope (CFM). (**A**) Schematic representation of the CFM. f and z are the measured values and f_d_, z_d_ are the desired values of force and position, respectively. (**B**) The CFM setup on an inverted microscope. The lower panels show an image of the MEMS-based force sensor on the left and a schematic on the right side. Scale bar = 1 mm. Images in (B) taken from [68].

A complete mechanical characterization of plant cells requires the application of multi-scale loads from nanonewtons to millinewtons, requirements that are satisfied by the cellular force microscope (CFM). The CFM is a mechatronic system, combining micropositioners and force-sensing devices with standard light microscopes. The core of the system is a microelectromechanical systems (MEMS)-based capacitive microforce sensor [72], which can be equipped with probes of different geometries, depending on whether global or local properties are to be characterized. Tungsten probes with diameters ranging from 100 nm to 5 µm and lengths ranging from micrometers to millimeters are available. Extensions with various geometries (e.g., spheres or pyramids) can also be attached to the sensor tip. The force sensor is mounted on a three-axis positioning system to allow fast and precise positioning over a range of 27 mm with a resolution of 5 nm. The entire system is integrated with an inverted light microscope, to which a CCD camera is attached to facilitate the initial positioning of the sensor probe and to follow the course of the experiment. A schematic representation of the CFM is shown in Figure 1.

The CFM was first described by Felekis and colleagues. SI-traceable calibration and thorough uncertainty analysis characterization make the MEMS microforce sensors an ideal tool for the investigation of the micromechanical properties of living plant cells [73]. A careful study of epidermal cells from onion peel revealed the influence of turgor pressure and indentation angle on CFM measurements. The large travel range of the positioning system allowed raster scans on large areas that cover several cell files, resulting in combined topography and stiffness maps. Only the use of long, thin probes made it possible to assess the depressions between two adjacent cells. By using an FEM-based modeling approach, the authors could identify local differences in mechanical cell wall properties [67].

CFM experiments on growing lily pollen tubes confirmed previous results, which described that the tip of the pollen tube appears to be softer than the more distal regions. At first glance, this agrees well with the fact that the biochemical composition of the cell wall in the apical region is different from that in the rest of the pollen tube. At the tip, pectins are methylesterified, whereas in the shank, they become cross-linked by calcium (Ca^2+^). Crystalline cellulose and callose are absent from the apical region. Based on these differences, it was concluded that the tip region of the pollen tube must be softer than the shank, which made sense, since growth occurs exclusively in the tip region. Vogler and colleagues [68] combined the CFM measurements with an FEM-based modeling approach, which allowed them to extract the Young’s modulus of the cell wall, as well as turgor pressure, using data on the apparent stiffness and shrinkage of the pollen tube upon plasmolysis, respectively. For the FEM model, uniform material properties were assumed, *i.e.*, no differences between pollen tube tip and shank. Nevertheless, the simulated apparent stiffness curves closely reflected the measurements, showing lower apparent stiffness in the tip region. This means that the observed decrease in stiffness towards the tip can be explained by the geometry of the pollen tube alone and, despite biochemical differences in composition, is not dependent on different mechanical properties of the cell wall material in tip and shank.

The influence of cell geometry on the apparent stiffness measurements illustrates one of the problems arising in CFM experiments or indentation experiments in general. Since available sensors are only capable of measuring forces parallel to the axis of the probe, every contact angle between the sample and the sensor probe >0 will affect the measured forces and, thus, the apparent stiffness [15]. Therefore, it will be important to produce 2D sensors capable of measuring vertical and lateral forces at the same time or to develop systems that allow the rotation of either the sample or the sensor in order to measure normal to the cell wall surface.

The CFM has been instrumental in determining the effects of specific cell wall mutations on the mechanical properties of the wall. For instance, the *Arabidopsis xxt1 xxt2* double mutant displays a severe reduction in XyG. This leads to an altered morphology and, eventually, the bursting of pollen tubes. A very similar phenotype was found in the *xyloglucanase113* (*xeg113*) mutant. This mutant lacks functional extensins, which are a class of cell wall remodeling proteins. CFM measurements, in combination with FEM modeling, provided insights into the mechanical properties of such mutant pollen tubes. XyG deficiency weakened the cell wall, represented by a decrease in the elastic modulus (Young’s modulus) of the cell wall, while turgor pressure was unaffected in comparison to wild-type pollen tubes. In contrast, in the *xeg113* mutant, cell wall elasticity was not altered in comparison to the wild type, but turgor pressure was increased [36].

Another set of CFM experiments was performed to evaluate the effect of Ca^2+^ homeostasis on the apparent stiffness of *Arabidopsis* pollen tubes. Ca^2+^ homeostasis plays an important role in cell wall integrity. In an NADPH oxidase-deficient double mutant (*rbohH rbohJ*), which displays a pollen tube bursting phenotype, it was shown that the tip-focused Ca^2+^ gradient was dissipated [74,75]. CFM measurements revealed that *rbohH rbohJ* double mutants have a lower apparent stiffness than wild-type pollen tubes. Moreover, increasing the Ca^2+^ concentration in the growth medium reduced bursting and increased the apparent stiffness of the pollen tube to wild-type levels [76].

These findings exemplify that CFM data in combination with an FEM-based modeling approach is a tool that allows the precise characterization of the fine-tuned interplay between turgor pressure as a driving force of growth and the flexibility of the cell wall to control cell extension and expansion.

## 6. Real-Time Cellular Force Microscopy

Due to its flexibility, the CFM can be used to study the mechanical properties of multiscale biological specimens. However, taking measurements of fast growing cells is tedious. For example, following the growing tip of a pollen tube to measure mechanical cell wall properties at the same position over time requires manual positioning of the sensor probe for every single measurement, which also includes the repositioning of the microscope stage. Because of the variation between biological specimens, when measuring their mechanical properties, it is very important to measure a high number of samples in a given experiment. Furthermore, the goal is not only to measure, but also to correlate mechanical properties with phenotypes among different cell wall mutants. When cells with either a limited lifetime or rapid change of their mechanical properties are studied, high throughput and speed is required.

Automation is a key factor for high-throughput experiments, and to this end, the real-time CFM (RT-CFM) was developed [77,78]. By automating procedures, such as sample selection and mechanical loading, the experiment time is drastically reduced, which allows hundreds of measurements a day, whereas the CFM is limited to a few tens of measurements. For the sample selection routine, computer vision techniques were employed, which allow the user to define an area of interest (AoI) of the sample. The RT-CFM automatically creates an interactive image based on overlapping snapshots of the AoI. On the resulting interactive image, the user then selects single point(s), a mesh of points with defined characteristics (number of points, point-to-point distance), an arbitrary area along with a number of points, a point-to-point distance or a combination of the above. The exact measurement points are calculated, and the sensor tip is positioned automatically at each point using visual servoing techniques (vision-based robot control). A computer vision algorithm calculates the image distance between the sensor location and the target point and transforms this distance to positioner coordinates, and the sample is moved until the image distance is minimized.

Moreover, a functionality for cell tracking is included in the RT-CFM. The output of the tracking module can be a distinctive feature of a cell, e.g., the tip of a growing pollen tube. The coordinates of the tracked feature can be used as input to the visual servoing module, and thus, measurements can be performed at a localized point with respect to the feature. This functionality is very useful, for instance for conducting repeated measurements at a given distance from the pollen tube tip.

A further improvement of the RT-CFM lies in its positioning capabilities and acquisition architecture. The experiments with the CFM revealed a need for sub-micrometer positioning with minimum errors, due to the size of the cells being at the microscale. Thus, a dual-stage configuration is used that consists of a combination of positioners mounted on top of each other. In the dual stage, a coarse XY positioner for large range, but slow and low-resolution movements is used, integrated with a fine XYZ positioner for fast and high-resolution, but small range movements. The dual-stage configuration benefits from the advantages of both positioners and achieves a position resolution of 2 nm over a range of 25 mm. With the parallel kinematic design of the current system, cumulative guiding errors and backlash are avoided. At the same time, non-linearities, poor accuracy and off-axis motions are compensated for by a parallel metrology approach [77,78].

**Figure 2:**
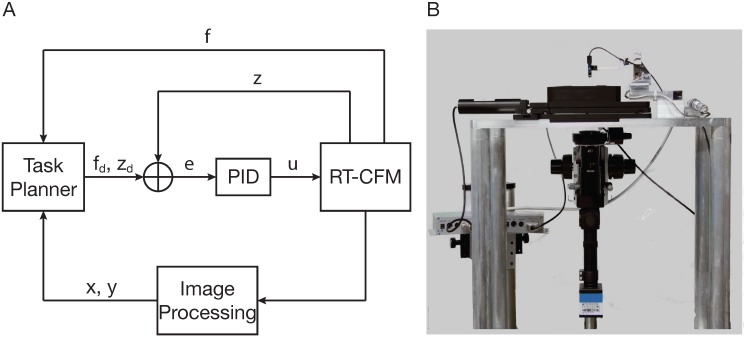
The real-time cellular force microscope (RT-CFM). (**A**) Schematic representation of the RT-CFM. f and z are the measured values, and f_d_, z_d_ are the desired values of force and position, respectively. e is the error between the desired and the measured values of force and position, and u is the control signal that is fed into the RT-CFM positioner. x and y are the coordinates of the measurement location to which the system has to be positioned. The task planner switches the system between measurement, positioning, calibration and post-processing tasks. The proportional-integral-derivative (PID) is the control scheme used to control to the positioner. (**B**) Custom inverted RT-CFM setup. Block diagram in (A) adapted from [78].

The actuation control input and the position readout in the RT-CFM are interfaced with a real-time computer. This configuration allows one to acquire real-time, deterministic experimental data and implement real-time control with force and position feedback. Due to the faster dynamics of the piezoelectric stack actuators in combination with the real-time control, the execution time required for experiments is drastically reduced, allowing for faster measurements. A schematic representation of the RT-CFM is shown in Figure 2.

The abilities of the RT-CFM were demonstrated by characterizing the apparent stiffness over the entire area of a pollen tube as it grows, in contrast to measurements along the pollen tube longitudinal axis that have been previously obtained [68,73,79]. Felekis and colleagues took measurements over an area of 60 µm × 50 µm, starting at the pollen tube tip with maximum forces reaching 1.5 µN. The measured stiffness maps were combined with topography maps of growing pollen tubes. To study the effect of cell wall growth on the mechanical properties of the cell wall, a 14 µm × 5 µm area on the pollen tube was measured at three different time points during its growth. The visual servoing functionality allowed the accurate localization of the sensor tip on the growing cell and the comparison of successive experiments. The experimental time was 30 seconds and the time interval between experiments 2.5 minutes [77].

In addition to the mechanical characterization of the pollen tube, it would be interesting to understand how external mechanical stresses are perceived, influence behavior and are, in turn, modified by the cell. In combination with fluorescence microscopy, the RT-CFM is a powerful tool for quantifying the physiological responses of cells to mechanical stimulation. For instance, Felekis and colleagues applied precise mechanical loading while monitoring the changes in intracellular Ca^2+^ levels [77].

## 7. Outlook

The RT-CFM is a versatile, highly automated system, which allows high-throughput mechanical measurements on growing cells. In combination with fluorescence microscopy, it will allow well-defined mechanical stresses to be applied to individual cells and the physiological responses to be followed in real time. Such a setup would, for example, allow mechanical triggers to be set in different parts of growing pollen tubes that express a Ca^2+^-sensing construct and observe the effects on the homeostasis of intracellular Ca^2+^. Moreover, RT-CFM has the potential to be used in combination with multi-well plates to compare different growth conditions in parallel or in combination with microfluidic channels, which would allow researchers to manipulate conditions and measure the changes in real time.

Despite the advantages of the CFM and RT-CFM, there are remaining problems that need to be addressed. It is not possible to measure cell wall stiffness directly due to the influence of turgor pressure. FEM-based models are required to extract the Young’s modulus and turgor pressure from the measured apparent stiffness values, which involves the measurement of additional parameters, such as pollen tube geometry and cell wall thickness. Another disadvantage is that measurements can only be taken perpendicular to the cell surface, and thus, it is not possible to get data from curved regions of cells, for example the pollen tube tip. This problem could be overcome by the development of 2D-MEMS force sensors, which allow force measurements along the Z- and X-axis. While prototypes of such 2D-MEMS force sensors exist [80,81], they are not yet commercially available.

Although not yet applied to living cells, further technological developments, such as multi-frequency atomic force microscopy (MF-AFM), will open completely new, exciting possibilities [82,83,84,85]. Improved cell wall models based on CFM or RT-CFM data can be used to identify regions where rapid changes in stiffness parameters occur. The application of MF-AFM on these areas will deliver high resolution data for cell topography, force, elasticity, dissipative energy loss as a measure of viscosity, and much more.

Another method with potential for studying biophysical parameters in plant cell growth is photoacoustic microscopy (PAM). It was successfully used to determine viscoelasticity in animal tissues, especially tumors [86,87]. With a lateral resolution of tens of microns, PAM was limited to measuring differences in viscoelasticity at the tissue level until recently. Newer setups, however, provide a resolution at the submicron level [88,89], which opens the possibility to precisely determine the subcellular regions where cell expansion occurs.
